# Effect of Ultrasonic Frequency on Structure and Corrosion Properties of Coating Formed on Magnesium Alloy via Plasma Electrolytic Oxidation

**DOI:** 10.3390/ma16155424

**Published:** 2023-08-02

**Authors:** Siti Fatimah, Farah Hazmatulhaq, Yujun Sheng, Tri Suhartono, Jeong Moo Oh, Nisa Nashrah, Jee-Hyun Kang, Young Gun Ko

**Affiliations:** School of Materials Science and Engineering, Yeungnam University, Gyeongsan 38541, Republic of Korea

**Keywords:** magnesium alloys, plasma electrolytic oxidation, ultrasonic frequency, corrosion

## Abstract

This study explores the application of ultrasonic vibration during plasma electrolytic oxidation (PEO) to enhance the corrosion resistance of magnesium (Mg) alloy. To this end, three different ultrasonic frequencies of 0, 40, and 135 kHz were utilized during PEO. In the presence of ultrasonic waves, the formation of a uniform and dense oxide layer on Mg alloys is facilitated. This is achieved through plasma softening, acoustic streaming, and improved mass transport for successful deposition and continuous reforming of the oxide layer. The oxide layer exhibits superior protective properties against corrosive environments due to the increase in compactness. Increasing ultrasonic frequency from 40 to 135 kHz, however, suppresses the optimum growth of the oxide layer due to the occurrence of super-soft plasma swarms, which results in a low coating thickness. The integration of ultrasonic vibration with PEO presents a promising avenue for practical implementation in industries seeking to enhance the corrosion protection of Mg alloys, manipulating microstructures and composition.

## 1. Introduction

In the recent decades, substantial research endeavors have been dedicated to the development of approaches aimed at enhancing both mechanical and corrosion properties of magnesium (Mg) alloys, which include techniques such as alloying, heat treatment, and surface modification [1,2]. Of these techniques, surface modification has gained increasing attention due to its ability to enhance the surface properties of Mg alloys without affecting their bulk performance. Plasma electrolytic oxidation (PEO) presents itself as a highly promising technique for surface modification, holding immense promise by forming a dense and thick oxide layer on the surface of Mg alloys and other valve metals (Al, Ti, Zr, and their alloys), which would improve their corrosion resistance and surface properties [3,4,5]. Despite its potential benefits, the PEO technique faces certain limitations, which includes a relatively low deposition efficiency and the occurrence of microcracks and micropores on the surface of the oxide layer [6,7]. Although post treatment by considering polymers has been reported to suppress the number of defects [8,9,10], such a strategy was hindered by two-step processing and would prolong the total fabrication duration.

Among various approaches to alleviate this problem, a one-step technique utilizing ultrasound technology exhibited a potential for collaboration with PEO, since it prevents particle agglomeration and promotes uniform surface finish with higher incorporated species [11,12,13]. Fathyunes et al. [14] documented that ultrasonic vibration improved the deposition efficiency of particles via electrochemical deposition and promoted the formation of a uniform and dense ceramic layer. Moreover, ultrasonic vibration modulated the formation of nanocrystalline and amorphous phases via the reduction–deposition process [15], which can further enhance the mechanical and corrosion properties of Mg alloys. Recently, Shen et al. [16] reported that the use of ultrasonic vibration on an Al 6061 alloy at 40 kHz during PEO increased the thickness of ceramic coating by ~20% within 10 min. The effect of microstructure on the electrochemical performance was not studied in their work. Moreover, although Ajiriyanto et al. [17] reported a similar tendency of increasing coating thickness for zircaloy-4 under the same frequency, the corrosion performance seemed to be similar to that without ultrasonic assistance. Despite these findings, the influence of ultrasonic assistance during the PEO process on the microstructure and corrosion resistance of Mg alloys remains comparatively underexplored and lacks comprehensive documentation, especially on the processing–microstructures–properties relationship.

On the other hand, the introduction of F^−^ ions into the oxide layer has been documented as a viable approach to enhance the anti-corrosion characteristics. This improvement is attributed to the ability of F^−^ ions, owing to their small size, to penetrate deeply into the coating thickness and effectively seal microdefects [18,19]. Such unique characteristics of F^−^ ions would be utilized to monitor the behavior of ultrasonic vibration during the incorporation of electrolyte species and formation of the oxide layer via PEO.

While it has been well accepted that ultrasonic vibration affected the deposition of particles, the study on the optimum ultrasonic frequency and the fundamental mechanism that would govern the nucleation and growth of the oxide layer remained less understood. Hence, the present study attempts to inspect the nucleation of the oxide layer influenced by ultrasonic vibration at different frequencies (0, 40, and 135 kHz) formed on the substrate at the onset of breakdown where the plasma discharge initiates. The use of F^−^ ions is to monitor the ionic transport behavior during incorporation assisted by ultrasonic vibration.

## 2. Materials and Methods

### 2.1. PEO Process

A plate of AZ31 Mg alloy with chemical composition of 3.08 wt.% Al, 0.76 wt.% Zn, 0.15 wt.% Mn, balanced Mg was utilized as the substrate material. Prior to PEO, the samples underwent mechanical polishing using #2400 SiC emery papers in distilled water, followed by rinsing with distilled water and ultrasonic cleaning in ethanol. A 20 kW AC power supply (ACP 1010, Gunpo, Republic of Korea) equipped with ultrasonic and cooling systems was used to perform PEO for 300 s with an applied current density of ~50 mA/cm^2^. A series of PEO coatings were carried out under AC condition at a frequency of 60 Hz. The electrolyte was prepared from concentrated K_3_PO_4_ (0.05 M), KOH (0.01 M), and lastly KF (0.007 M), which behave as compacting agent. To investigate the influence of ultrasonic frequency on the corrosion behavior of PEO-treated Mg alloys, an Ultrasonic Generator (Han Shin Tech 160 W, Anyang, Republic of Korea) with three different frequencies of 0, 40, and 135 kHz was employed, and the samples were referred to as U_f_0, U_f_40, and U_f_135, respectively.

### 2.2. Characterization

The surface morphologies and cross-sectional images of the oxide layer were investigated using a scanning electron microscope (SEM, Hitachi S-4800, Tokyo, Japan). The phase composition of the oxide layer was analyzed using X-ray diffraction (XRD Rigaku, D MAX-2500, Tokyo, Japan) with Cu Kα radiation, and the scans were performed with 0.02° step size in the 20~80° range. The plasma discharges on the surface of oxide layer were captured by digital camera (Canon EOS 700D, Tokyo, Japan) whilst the plasma intensity was analyzed using the image analyzer (ImageJ2, Fiji software).

### 2.3. Electrochemical Measurements

The electrochemical characteristics were assessed in a 3.5 wt.% NaCl solution, employing three distinct electrodes: a coated specimen with a 1 cm^2^ exposed area serving as the working electrode, a platinum plate utilized as the counter electrode, and an Ag/AgCl solution employed as the reference electrode. The corrosion properties of the oxide layer were evaluated using potentiodynamic polarization and electrochemical impedance test (Gamry Interface 1000, Philadelphia, PA, USA). The potentiodynamic polarization curves were measured from −0.25 to 0.4 V in respect to the open circuit potential (OCP) at a scan rate of 1 mV s^−1^ for approximately 20 min. In addition, EIS measurements were conducted at AC frequencies ranging from 1.10^6^ Hz to 0.1 Hz at an interval of 10 points per decade with 10 mV rms.

## 3. Results and Discussions

### 3.1. Transient Voltage Response and Nucleation of Plasma Discharges

**Figure 1:**
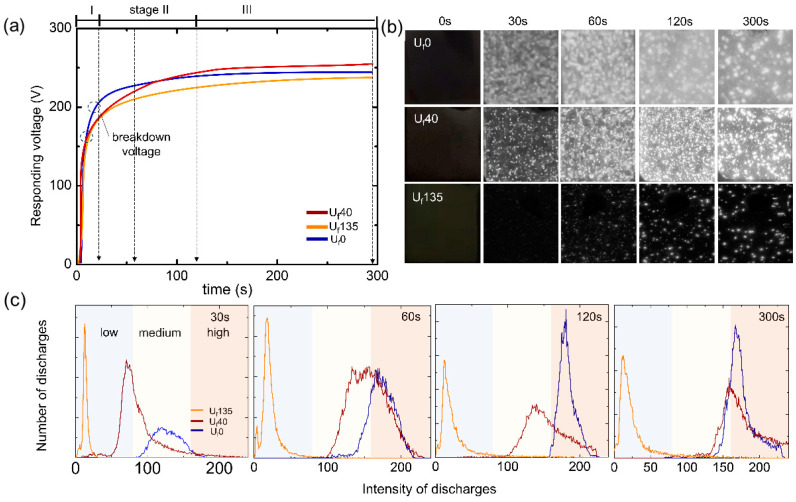
(**a**) Responding voltage vs. coating time curves of AZ31 Mg alloys processed via PEO in three different ultrasonic frequencies of 0, 40, and 135 kHz. (**b**) Evolution of plasma discharges with respect to coating time. (**c**) Simple histogram showing the fraction and intensity of plasma discharges using image analyzing software.

The effect of ultrasonic frequency on the behavior of plasma discharges was evaluated at the onset of breakdown and during the growth stage of the oxide layer by considering voltage response with respect to coating time, as shown in Figure 1. From the voltage–time curve, three stages of PEO were identified based on the increment of the corresponding voltage. Stage I was depicted by a sharp increase in slope as a passive film formed on the substrate by a conventional anodizing process obeying Ohm’s law [5]. The growth of the passive film persisted until reaching the breakdown voltage, which occurred when the applied potential surpassed the threshold for dielectric breakdown. Subsequently, plasma discharges were initiated, and the cell voltage experienced a gradual increase owing to the relatively consistent growth rate of the oxide layer. The region of decreasing slope after the breakdown voltage was denoted as stage II. The slope further decreased and showed almost plateau characteristics where the growth of the oxide layer was relatively constant, referred to as stage III. The initiation and the characteristics of plasma discharges, i.e., size, density, duration, and intensity, controlled the overall microstructures of the oxide layer via PEO [3]. Monitoring these characteristics during PEO is essential for understanding the final microstructures of the oxide layer without and with the assistance of ultrasonic vibration, along with the plasma discharges characteristic and the microstructures of the oxide layer.

From Figure 1, the breakdown voltage was recorded lower for those processed under ultrasonic vibration. Both U_f_40 and U_f_135 showed almost identical breakdown potentials of ~160 V, whilst that of U_f_0 was recorded as high as ~200 V. The reason for this phenomenon would be arising from the ultrasonic wave that generated cavitation and acoustic streaming which would facilitate the formation of weak spots for the initiation of plasma discharges through the dielectric breakdown of the insulator passive film to become conductive [20,21]. Acoustic streaming is the flow of fluid caused by sound propagation, leading to microstreaming effects [22]. This microstreaming enhances mass transport and fluid mixing, finding applications in drug delivery and industrial processes like particle dispersion and liquid cleaning. Microstreaming specifically refers to the streaming flow of fluid around an oscillating object, such as a gas bubble. The fluid flow is generated from the vorticity caused by the oscillation of the boundary layer surrounding, for example, an oscillating cavitation bubble.

When the ultrasound passed through the interface between the electrolyte and the oxide layer, the interface was heated up to a certain level, which would speed up the dissolution of the surface and induce weak spots (flaws/defects) [20,23]. A number of weak spots would allow the layer to be dielectrically broken down much easier to release plasma discharges [20,23]. It is noteworthy to note that the introduction of ultrasonic vibration resulted in a shorter duration for reaching the breakdown voltage. The time to reach the breakdown was recorded at 20 s for the U_f_40 and U_f_135 samples, whilst it was recorded at 30 s for U_f_0.

The responding voltage of U_f_40 was increased significantly in the middle of stage II, surpassing that of U_f_0, whilst U_f_135 was recorded as the lowest, despite having a higher slope than U_f_0. At the end of stage III, the responding voltage was recorded as the highest for U_f_40, followed by U_f_0 and U_f_135. It is widely known that the value of responding voltage would be related to the resistivity of the oxide layer [24]. Those results implied that the coating compactness/thickness would be in the order of U_f_40 > U_f_0 > U_f_135. Such a relationship will be discussed in Section 3.2.

Figure 1b shows optical images of plasma discharges during PEO of the samples U_f_0, U_f_40, and U_f_135. In general, the intensity of plasma discharges was found to be decreased when ultrasonic vibration was introduced during PEO. From Figure 1b, the upper (U_f_0) and middle (U_f_40) rows showed higher intensity of plasma discharges; whereas, the bottom row (U_f_135) showed less populated plasma discharges with relatively lower intensities. In the absence of ultrasonic vibration, it was clear that plasma discharges would manifest with non-uniform distribution, primarily concentrated in regions with high-intensity discharges. In contrast, U_f_40 showed relatively more homogeneous and milder intensity of discharges in all stages of PEO, implying the beneficial effect of ultrasonic vibration. By increasing the frequency of ultrasonic waves to 135 kHz, however, the characteristics of plasma discharges became too soft and less populated.

Figure 1c shows the plasma intensity and number of discharges from three different samples. It was clear that U_f_135 was populated by low-intensity discharges throughout the whole PEO process, whilst it was shifted from low- to medium- to high-intensity discharges. Such a phenomenon was believed to be associated with the strong obstruction by the high-frequency ultrasonic vibration of 135 kHz. At a low coating time (30 s), the surface of the substrate was predominantly occupied by plasma discharges of lower and medium intensities, evident from the discharges populated on both regions equally (Figure 1c). In particular, U_f_135 was populated by low-intensity discharges, U_f_0 was populated by medium-intensity discharges, and U_f_40 was dominated equally by both low and medium intensities of discharges. With increasing coating time to 60 s, the plasma intensities of U_f_40 and U_f_0 were shifted to medium-/high-intensity regions, where 40% of U_f_40 was dominated by medium-intensity regions. On the other hand, 75% of U_f_0 was dominated by high-intensity regions. At 120 s, the discharges of U_f_0 were identified as high-intensity discharges, whilst only ~40% of U_f_40 was identified as high-intensity discharges. When the duration of PEO was further prolonged to 300 s, U_f_0 was recorded to have the highest intensity and size of plasma discharges, as also evident from Figure 1b. Such plasma characteristics might be correlated with the features of an early-formed oxide layer, including the porosity and constitutive phases. From video imaging shown in Figure 1b, it was expected that the high intensity of plasma discharges in U_f_0 might not be suitable for obtaining a dense oxide layer due to the plasma discharges with rigorous behavior and destructive tendency.

### 3.2. Morphologies of the Oxide Layer

**Figure 2:**
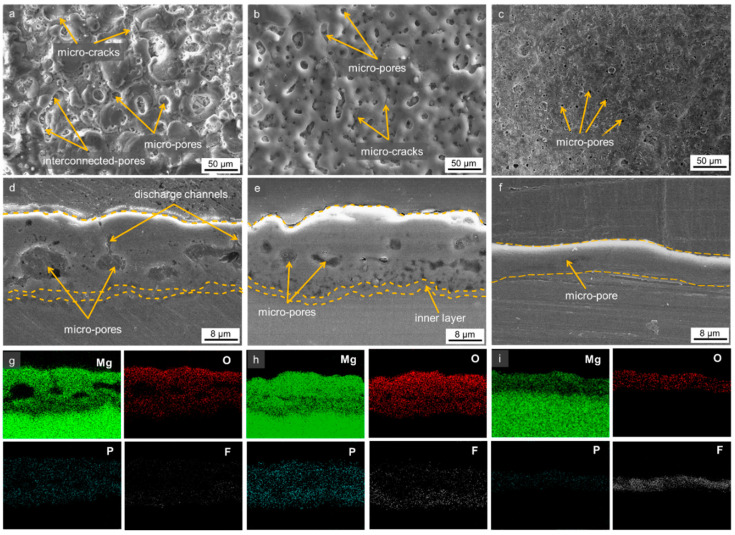
Surface morphologies and cross-sectional images of the oxide layer formed in three different ultrasonic frequencies of (**a**,**d**) 0, (**b**,**e**) 40, and (**c**,**f**) 135 kHz. EDS map area of Mg, O, P, and F elements on the surface of (**g**) U_f_0, (**h**) U_f_40, and (**i**) U_f_135.

Figure 2 shows surface morphologies and cross-sectional images of all samples along with EDS elemental mapping. Irrespective of the presence of ultrasonic waves, microstructures showed the typical porous surface of the PEO layer in all samples. With the introduction of ultrasonic vibration, the porosity and pore size decreased considerably with the increase in ultrasonic frequencies. The oxide layers of the U_f_0 and U_f_40 samples showed comparable thicknesses of ~15 ± 0.5 μm, whilst U_f_135 exhibited the lowest thickness of ~7 ± 0.2 μm. Interestingly, those which were fabricated with the assistance of ultrasonic vibration showed more compact/dense characteristics of the oxide layer. Although having the lowest thickness, U_f_135 exhibited the most compact coating characteristics, followed by U_f_40 and U_f_0. As documented earlier, the sample with a more dense coating would likely have better corrosion protection as compared to that having lower compactness [25,26]. Thus, it can be expected that U_f_40 would demonstrate better corrosion resistance than U_f_0. The main reason for the different coating thickness would stem from the presence of ultrasonic waves.

Since ultrasonic vibration generated the cavitation bubbles and acoustic streaming in the electrolyte, the collapse of the bubbles and flow of the streaming would help the deposition of the electrolyte species in addition to their assistance for promoting metal dissolution via local heating, hence accelerating coating growth [27]. Interestingly, although ultrasonic vibration assisted the growth of the oxide layer, the final coating thickness of U_f_135 was found to be the thinnest, suggesting that different mechanisms might govern the growth rate of the oxide layer. Taking into account the characteristics of soft plasma discharges in the U_f_135 sample, it can be inferred that soft plasma discharges might not effectively assist the growth rate but exhibit the impact on enhancing its compactness. Recent investigation in the field of fluid physics suggested that despite the finer size of cavitation bubbles and highest microstreaming tendency in the system with high-ultrasonic frequency, the released energy once cavitation bubbles imploded was found to be higher in low-frequency waves [28]. Therefore, the surface of the substrate would receive higher impact and weak spots which have lower electrical resistance suitable for the initiation of plasma discharges. As a consequence, a greater number of plasma discharges would be observed enveloping the surface of the substrate. On the other hand, the energy in the system with higher frequency might be exchanged for the continuous mixing, agitation, and incorporating of particles during PEO.

Figure 2g–i display EDS elemental maps taken from the cross-sectional images from Figure 2d–f, comprising Mg, O, P, and F elements. The F elements were found to be incorporated more abundantly (in wt.%) with homogeneous behavior in the presence of ultrasonic vibration and with higher ultrasonic frequency (sample U_f_135). This is because the size of F^−^ ions is small while electronegativity is high, so it would penetrate deeper into the oxide layer [18]. Moreover, the incorporation of F^−^ ions was made throughout the coating thickness of the U_f_135 sample, which was probably due to the steady mixture of electrolyte species with the aid of ultrasonic vibration. The elemental compositions of the oxide layers are listed in Table 1. The results confirmed that the F element was incorporated more effectively in the presence of ultrasonic vibration with a high frequency.

### 3.3. Compositional Analysis of the Oxide Layer

**Figure 3:**
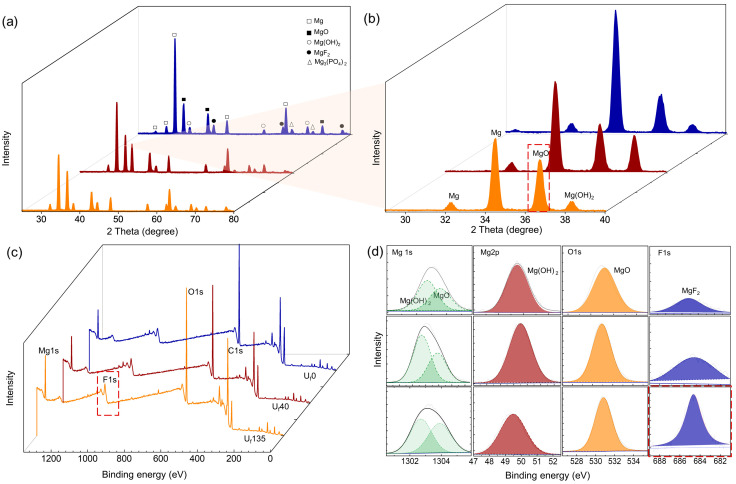
(**a**) XRD pattern of AZ31 Mg alloys processed via PEO in three different ultrasonic frequencies of 0, 40, and 135 kHz. (**b**) High magnification of XRD peaks in the range of 20 to 40 2 Theta degree showing relative fraction of Mg, MgO, and Mg(OH)_2_ representative peaks. (**c**) XPS survey of all samples. (**d**) XPS analysis of Mg1s, Mg2p, O1s, and F1s from all samples.

Figure 3a,b show the constitutive compounds that are present in samples U_f_0, U_f_40, and U_f_135. The XRD peaks were mainly composed of MgO (#JCPDS No. 78-0430) and Mg(OH)_2_ (#JCPDS No. 07-0239), with secondary peaks of Mg_3_(PO_4_)_2_ (#JCPDS No. 33-0876) and MgF_2_ (#JCPDS No. 41-1443). In addition, the Mg peaks were detected due to the X-ray deep penetration into the Mg substrate. Although the thicknesses of U_f_0 and U_f_40 were similar, the detection of MgO and Mg(OH)_2_ was found to be higher in the U_f_40 sample (Figure 3b), indicating a higher density of the oxide layer as a result of a more massive incorporation of electrolyte species. The results verified that the ultrasonic wave with suitable frequency (~40 kHz) would facilitate the transport of ions from the electrolyte and their incorporation, which further led to the generation of a dense and thick oxide layer. Strikingly, the high percentage of MgO was found in sample U_f_135, higher than that found in U_f_40, which implied that several fractions of Mg(OH)_2_, which was the earlier phase of oxide layer composition, transformed into MgO via the dehydration process. It is suggested that the dehydration was facilitated by the high amount of energy found in the system with a higher frequency (135 kHz).

Figure 3c shows the surface chemistry of the oxide layer represented by XPS survey peaks comprising Mg, O, P, and F elements. In general, the intensity of both Mg and O were found to be higher in the U_f_40 sample as compared to the other samples, indicating that the oxide layer of U_f_40 possessed the highest mass-to-volume ratio. Whilst the percentages of the P and F elements were comparable between U_f_0 and U_f_40, the intensity of the F element, however, increased with the increase in the frequency of ultrasonic vibration, implying that the ultrasonic vibration would facilitate the incorporation of particles. In line with EDS results, the F element was detected homogeneously throughout the coating thickness, implying that homogeneous incorporation was established from the initial stage of the formation of the oxide layer. Table 2 shows the processing phenomena during PEO and the characteristics of the resultant oxide layer. From Table 2, it can be inferred that the characteristics of plasma discharges and ultrasonic behavior during PEO would significantly affect the characteristics of the oxide layers. For example, samples which displayed more populated and moderate intensity of plasma discharges would possess thick and relatively dense oxide layers.

### 3.4. Corrosion Protection Capabilities of the Oxide Layer

**Figure 4:**
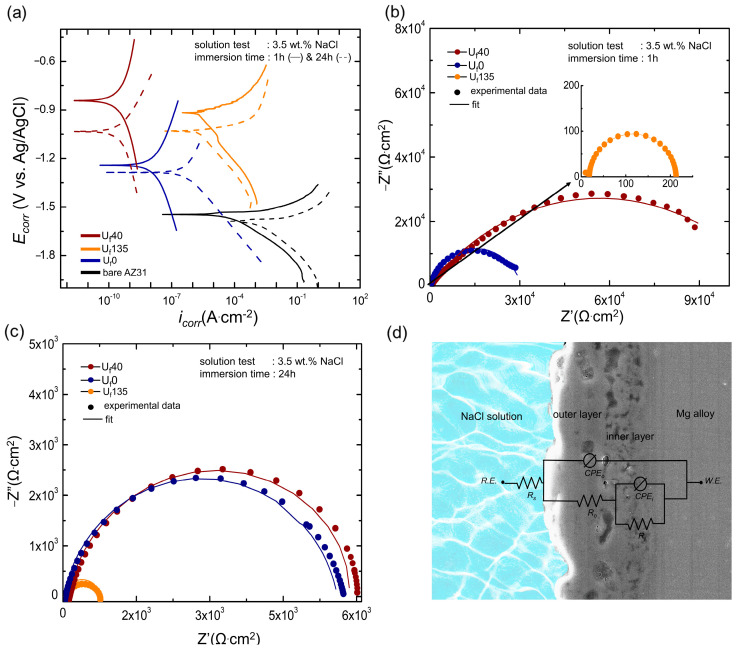
(**a**) Potentiodynamic polarization curves of all samples after immersion in 3.5 wt.% NaCl for 1 and 24 h. It is shown that bath U_f_40 sample showed better corrosion protection. EIS Nyquist plots of all samples after submerged in 3.5 wt.% NaCl for (**b**) 1 h and (**c**) 24 h, calculated from 106 to 0.1 Hz. The listed values were obtained based on the equivalent circuit model shown in (**d**).

Figure 4a shows polarization curves of all samples after immersion in the test solution for 1 and 24 h. The corrosion potential (*E_corr_*), corrosion current density (*i_corr_*), and Tafel slopes (*β_a_*/*β_c_*) for anodic/cathodic branches were obtained from the Tafel extrapolation whilst the polarization resistance (*R_p_*) values were estimated using the Stern–Geary equation, as shown in Equation (1) [29], and the results are listed in Table 3.

(1) $$R_{p}=\frac{\beta_{a}\cdot \beta_{c}}{{2.303\;i_{corr}(\beta }_{a}+\beta_{c})}$$

From Figure 4a, it could be inferred that *E_corr_* of the PEO-treated AZ31 Mg alloy shifted to a more noble region when the ultrasonic wave was introduced, whilst *i_corr_* shifted to the left with a lower corrosion rate from U_f_0 to U_f_40. The *E_corr_* would be in the order of U_f_40 > U_f_135 > U_f_0, whilst *i_corr_* would be in the order of U_f_40 > U_f_0 > U_f_135. The low *E_corr_* value in U_f_0, which indicated lower stability of the oxide layer upon contact with the test electrolyte, was presumably due to the presence of connected micropores, providing short access for corrosive ions to reach the metal substrate. In the case of U_f_135, the high amounts of MgO and MgF_2_ having higher oxidation potential than Mg(OH)_2_ would be responsible for its relatively high *E_corr_* value [30,31].

The *i_corr_* values would be strongly correlated with the thickness/compactness of the oxide layers, where U_f_40 having a higher coating compactness than U_f_0 exhibited better anti-corrosion resistance. Interestingly, despite U_f_135 demonstrating a higher compactness than that of U_f_0, the results showed that U_f_0 exhibited better anti-corrosion performance. Hence, the role of thickness in the present work was more dominant than the role of compactness against corrosion inhibition. The increase in the *R_p_* value of the sample processed with the assistance of ultrasonic vibration at 40 kHz was about two orders of magnitude from that without sonification. On the contrary, the *R_p_* value decreased as the ultrasonic frequency was elevated to 135 kHz, which would be correlated with a low coating thickness despite a considerable increment in the coating compactness.

To conduct a more systematic comparison of the electrochemical behavior, the impedance data acquired by EIS (Figure 4b,c) were analyzed using equivalent circuit (EQC) model, as shown in Figure 4d. The impedance responses obtained from the data indicated that the electrochemical behavior of the samples coated via PEO could be represented by analogous circuit components [32]. In general, the EIS data were parallel to that of polarization data, where a higher radius of capacitive semicircles demonstrated a higher resistance toward corrosion.

In EQC models, *R_o_* and *R_i_* denote the outer and inner parts of the oxide layer, respectively, whereas *R_s_* denotes the resistance of the electrolyte in contact with the oxide layer surface. *CPE_o_* and *CPE_i_* denote the respective constant phase elements of the outer and inner layers. The impedance value of *CPE* (*Z_CPE_*) was defined by Equation (2) [33], as follows:

(2) $$Z_{CPE}=1/[Y{\left(j\omega \right)}^{n}]$$

where *ω* was the angular frequency, *j* was the imaginary number, *Y* and *n* were *CPE* parameters. Those parameters described the insulation tendency occurring on the electrolyte/oxide interface where the *n* value reflected the degree of surface homogeneity. Between the experimental data and the EQC model provided in Table 3, a satisfactory match was documented. The *R_s_* values were equivalent for all the samples because they were tested on the same electrolyte even when the test was prolonged to 24 h. The existence of overlapping semicircles in the Nyquist plot indicates that the parallel combination of two R–C time constants, which represent the inner and outer layers, suggested the absence of clear boundaries between them. This indicated that the discharge channel allows the corrosive medium to reach the inner layer via shorter paths. Consequently, portions of both the inner and outer layers exhibit similar electrochemical characteristics, as evidenced by the overlapping semicircles in the plot.

In general, EIS data agreed well with the results of the potentiodynamic polarization tests. The slight difference in magnitudes between polarization and EIS data was common because the potentiodynamic polarization test provides a more immediate method and did not consider the amount of corrosion product that would be anticipated from the comparatively gradual process of immersion corrosion in EIS. Nevertheless, they both showed a similar tendency where U_f_40 showed the best corrosion resistance followed by U_f_0 and U_f_135.

The corrosion resistance of the U_f_40 sample was the best among the others in both short and long immersion durations. Within an immersion time of 1 h, the values of *R_o_* and *R_i_* were in the order of U_f_40 > U_f_0 > U_f_135. It was clear that the order aligned with the characteristics of compactness and thickness of the oxide layer, as shown in Figure 2. Such a phenomenon was also supported by the *n* values shown in Table 4, which represent the homogeneity level of the layer. The *n* values of U_f_135 displayed values close to one (*n_o_* = 0.95, *n_i_* = 0.99) for both inner and outer layer regions, confirming the high microstructural homogeneity throughout the coating thickness. The results implied that the soft and short duration of plasma discharges played an important role in the formation of a compact oxide layer.

When the resistances of *R_i_* and *R_o_* were compared, it was found that *R_i_* values were consistently higher than those of *R_o_* in all conditions. This dominance of the inner layer in terms of resistance over the outer layer was also noted in previous studies of the PEO oxide layer [3]. From the discussions presented above, it could be proposed that the introduction of ultrasonic vibration induces the formation of soft plasma discharges similarly. The growth of the oxide layer would be maintained by electrochemical reactions even though the plasma intensity would be decreased. In addition, the F^−^ ions incorporated in the oxide layer might impede the development of discharge channels throughout the coating thickness. This effect together with the plasma softening accounts for the dense microstructure and excellent corrosion resistance of the oxide layer formed in the electrolyte with the assistance of ultrasonic vibration.

### 3.5. Formation Mechanism of the Oxide Layer under the Interference of Ultrasonic Wave

**Figure 5:**
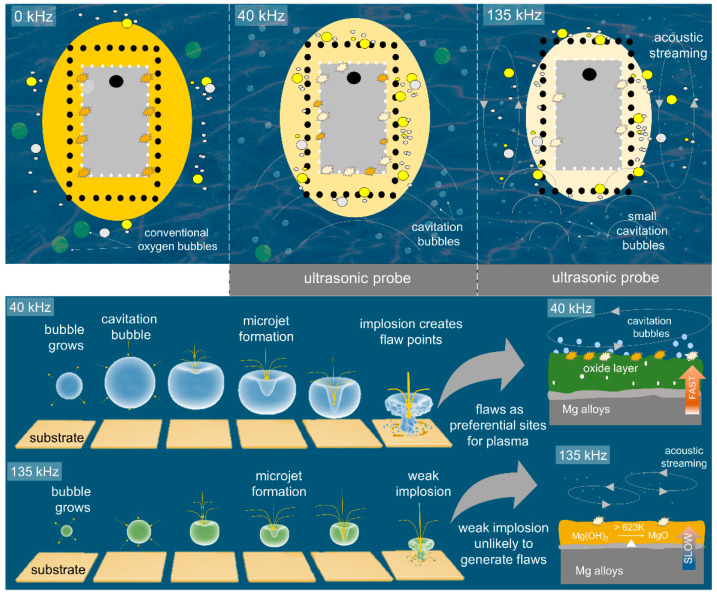
Schematic figure to illustrate the formation mechanism of the oxide layer under different ultrasonic frequencies of 0, 40, and 135 kHz. The utilization of ultrasonic frequency of 40 kHz generated strong cavitation bubbles suitable for triggering plasma discharges and incorporating electrolyte species which led to faster coating growth.

The schematic illustration in Figure 5 was drawn to suggest the nucleation and growth mechanisms of the oxide layer under the assistance of ultrasonic vibration. The ultrasonic wave propagates through the liquid medium, creating alternating regions of compression and rarefaction, regions of low and high pressure. The wave speed is linearly proportional to the wave frequency, as shown in Equation (3) below:

(3) v=f×λ

where *v* is the wave speed, f is the frequency, and λ is the wavelength. Therefore, the wave speed of fluid in the electrolyte with high ultrasonic frequency (135 kHz) would have a higher value than those with lower frequencies.

Ultrasonic waves promoted continuous mixing and diffusion of the electrolyte solution near the electrolyte–substrate interface and indirectly facilitated the incorporation of electrolyte species. This increased mixing and diffusion resulting from ultrasonic agitation promotes a uniform distribution of the electrolyte throughout the bulk solution, enabling better contact and interaction between the electrolyte and the substrate by enhancing mass transport [34,35,36]. Low-frequency ultrasonic waves, such as 40 kHz, have longer wavelengths and penetrate deeper into the substrate materials to seal microdefects. In contrast, high-frequency ultrasonic vibration, however, is more likely to be absorbed or scattered by surfaces and tends to have limited penetration into solid objects [37]. Therefore, we suggested that the incorporation of electrolyte species in U_f_135 occurred simultaneously with coating growth to ensure homogeneous distribution.

When ultrasonic waves passed through a liquid medium, they created high-frequency vibrations that caused the liquid to generate cavitation bubbles [20]. Cavitation refers to the formation, growth, and collapse of tiny bubbles in the liquid, as shown in the bottom part of Figure 5.

According to Poulain et al. [38], the flow velocity of cavitation bubbles was postulated by considering the size of the bubble, *R_b_*, as expressed by Equation (4) below:

(4) $$u\left(r,t\right)={\left(\frac{R_{b}\left(t\right)}{r}\right)}^{2}{\dot{R}}_{b}(t)$$

Furthermore, the internal energy of the bubble could be defined in the following manner, as shown by Equation (5) [39,40]:

(5) $${\Delta E}_{i}=-p\left(t\right)\cdot \Delta V\left(t\right)+4\pi \;r^{2}\frac{\Delta t}{M_{H_{2}O}}\dot{m}C_{V,\;H_{2}O}T+4\pi \;r^{2}\Delta t\left(\frac{T_{liq}}{L_{th}}\right)$$

where *p*(*t*) is the internal bubble pressure; Δ*V*(*t*) is the volume difference; $M_{H_{2}O}$ is the water molar mass; $C_{V,\;H_{2}O}$ is the heat capacity of water; *T*_*liq*_ is the temperature of the external bubble surface; and *L*_*th*_ is the thickness of the thermal boundary layer.

The collapse of these bubbles produced localized high pressures and temperatures, generated microstreaming and agitation within the liquid which would enhance mixing, promoted mass transfer, and facilitated the interaction between the liquid and solid surfaces [41]. More importantly, the collapse of cavitation bubbles created weak points on the impacted surface and acted as the preferential sites for the nucleation of plasma discharges. As can be seen from Figure S1 in Supplementary Materials, the size of cavitation bubbles decreased as ultrasonic frequency increased from 40 to 135 kHz. Due to the smaller size of the bubbles, the collapsing bubbles released insufficiently low energy to create weak points. Therefore, a lower population of plasma discharges was observed on the surface of U_f_135 during PEO. Accordingly, the growth of the oxide layer will be slower as compared to that of the U_f_40 and U_f_0 samples.

High-frequency ultrasonic vibration of 135 kHz generally provided more concentrated energy due to the shorter wavelength [42,43]. Low-frequency ultrasonic vibrations such as 40 kHz, on the contrary, tended to create larger and more pronounced streaming patterns in the liquid [42,43]. These larger-scale streaming patterns resulted in a broader coverage area and a greater chance for particles to be transported toward surfaces or boundaries for deposition. Although high-frequency ultrasonic vibrations generate more intense microstreaming effects, it does not necessarily translate into faster coating growth. The higher intensity of microstreaming at 135 kHz may displace particles or keep them suspended for longer periods in the electrolyte, making it more difficult for them to settle and deposit onto the surface. On the other hand, low frequency ultrasonication (40 kHz) typically induced larger and more stable cavitation bubbles. These cavitation bubbles created stronger localized fluid flaws and contributed to particle movement and deposition [20,23,43]. Such phenomena might be the reason for the massive deposition of electrolyte species, mainly (hydr)oxides and metal ions on the surface of U_f_40.

High frequency of ultrasonic vibration (135 kHz) tends to produce smaller and more transient cavitation bubbles, which may have a comparatively less influence on the generation of weak spots for plasma ignition and/or particle deposition [43]. High-frequency ultrasonic waves (135 kHz), experience greater attenuation in liquid compared to that of lower frequencies. This attenuation reduced the propagation range of the ultrasonic waves, limiting the effective reach of the microstreaming and cavitation effects. In contrast, a lower frequency might propagate further and cover a larger volume, increasing the chance of particle deposition. Therefore, more (hydr)oxides and metal ion species would be deposited and foster the growth of the PEO layer on the U_f_40 sample.

The present study suggested that an excellent combination would be achieved between compactness and coating thickness through the utilization of PEO assisted by ultrasonic vibration at a low frequency of 40 kHz. As the outcomes of this study are predominantly qualitative, additional exploration is necessary to establish a solid theoretical foundation using a quantitative methodology. This is crucial in comprehending the correlation between ultrasonic frequency/energy and the attributes of interface phenomena (such as cavitation bubbles and acoustic streaming), plasma discharges (including size, intensity, and distribution), and their subsequent impact on microstructures and electrochemical performances. A dedicated collaboration from various research fields, entailing plasma physics, electrochemistry, electrical engineering, etc., could help to obtain a comprehensive picture of the system.

## 4. Conclusions

The present investigation unveils insights into the impact of ultrasonic frequency on the microstructure and properties of the oxide layer, accounting for the intricate interplay between plasma sizes, cavitation bubbles, and other pertinent characteristics. The following is a summary of the findings.

1. Plasma swarm appeared later at a lower voltage when ultrasonic vibration was introduced to the system during PEO due to the presence of collapsing cavitation bubbles which created flaws for the initiation of dielectric breakdown.
2. The density of plasma discharges increased while their average size decreased with the use of ultrasonic vibration, thanks to ultrasonic waves which created homogeneous mixing through agitation, acoustic streaming, and cavitation bubbles which led to collision and plasma softening.
3. The ultrasonic vibration induced the formation of a dense oxide layer by virtue of softened plasma characteristics and homogeneous incorporation of F^−^ ions throughout coating thickness.
4. A trade-off between thickness and compactness appeared when a high ultrasonic frequency of 135 kHz was employed, which induced a decrease in corrosion resistance. While the high frequency allowed for enhanced compactness, it came at the expense of reduced thickness, which in turn had a negative impact on the material’s ability to withstand corrosion.

## Figures and Tables

**Table 1:** EDS results of the PEO-treated AZ31 Mg alloy in three different sonification frequencies: 0, 40, and 135 kHz. Area for analysis is taken from Figure 2.

Sample	Mg (wt.%)	O (wt.%)	P (wt.%)	F (wt.%)
U_f_0	23.26 ± 1.30	68.28 ± 1.80	5.41 ± 1.33	3.05 ± 0.53
U_f_40	22.39 ± 1.14	68.79 ± 1.22	5.42 ± 0.91	3.40 ± 0.61
U_f_135	21.10 ± 0.51	69.98 ± 0.73	2.74 ± 0.40	6.18 ± 0.22

**Table 2:** General characteristics of PEO-treated AZ31 Mg alloy in three different ultrasonication frequencies of 0, 40, and 135 kHz.

Sample	Plasma Intensity	Microdefects			Passivation Layer		
		Pores Size (Μm)	Porosity (%)	Cracks	Thickness (Μm)	Growth Rate (Nm/S)	Percentage of F Particles
U_f_0	bright	6.25 ± 0.72	19.38	many	15.10 ± 0.53	50.34 ± 0.52	low
U_f_40	moderate	4.69 ± 0.54	14.83	few	15.41 ± 0.33	51.30 ± 0.31	medium
U_f_135	dim	2.01 ± 0.21	2.05	scarce	7.32 ± 0.24	24.33 ± 0.12	high

**Table 3:** Polarization resistance of all samples. The polarization tests were performed in 3.5 wt.% NaCl solution measured from –0.25 to 0.4 V vs. open circuit potential. The immersion time was made for 1 and 24 h.

Immersion Time	Sample	*i_corr_* (A·cm^2^)	*E_corr_* (V)	*β_a_* (V/Decade)	*β_c_* (V/Decade)	*R_p_* (Ωcm^2^)
1 h	bare AZ31	1.20 × 10^−5^	−1.51	−0.05	0.16	1.36 × 10^3^
	U_f_0	2.39 × 10^−8^	−1.24	−0.43	0.47	4.06 × 10^6^
	U_f_40	1.26 × 10^−9^	−0.84	−0.11	0.15	2.24 × 10^7^
	U_f_135	2.80 × 10^−6^	−1.00	−0.04	0.19	5.34 × 10^3^
24 h	bare AZ31	5.25 × 10^−3^	−1.54	−0.04	0.12	2.62 × 10^1^
	U_f_0	2.48 × 10^−7^	−1.29	−0.31	0.16	1.85 × 10^5^
	U_f_40	5.47 × 10^−10^	−1.03	−0.27	0.63	1.51 × 10^8^
	U_f_135	2.40 × 10^−5^	−1.12	−0.02	0.16	3.75 × 10^2^

**Table 4:** Electrochemical impedance parameters of PEO-treated AZ31 Mg alloy. The impedance tests were measured in brine solution (3.5 wt.% NaCl) under the frequency range of 10^6^ to 0.1 Hz. With the equivalent circuit model shown in Figure 4d, the values are produced iteratively.

Immersion Time	Sample	R_s_ (Ω·cm^2^)	*R_o_* (Ω·cm^2^)	*R_i_* (Ω·cm^2^)	*n_o_*	*CPE_o_* (S.s^n^.cm^−2^)	*n_i_*	*CPE_i_* (S.s^n^.cm^−2^)
1 h	U_f_0	17.32	3.94 × 10^3^	3.35 × 10^4^	0.58	4.17 × 10^−6^	0.87	3.81 × 10^−6^
	U_f_40	19.50	1.35 × 10^5^	1.14 × 10^5^	0.56	1.99 × 10^−8^	0.85	3.84 × 10^−6^
	U_f_135	18.31	1.77 × 10^2^	2.13 × 10^2^	0.95	9.62 × 10^−9^	0.99	4.87 × 10^−5^
24 h	U_f_0	19.74	5.58 × 10^3^	1.50 × 10^4^	0.73	8.24 × 10^−3^	0.95	8.43 × 10^−7^
	U_f_40	20.23	1.06 × 10^5^	1.05 × 10^5^	0.87	3.73 × 10^−7^	0.96	5.91 × 10^−5^
	U_f_135	22.50	2.03 × 10^2^	5.36 × 10^2^	0.92	1.03 × 10^−5^	0.98	1.70 × 10^−2^

## Data Availability

The data presented in this study are available in Supplementary Materials.

## Supplementary Materials

The following supporting information can be downloaded at: https://www.mdpi.com/article/10.3390/ma16155424/s1. Figure S1. Optical images showing the plasma discharges appearance during PEO process under ultrasonic frequency of (a) 0, (b) 40, and (c) 135 kHz, respectively. Unlike conventional PEO, ultrasonic-assisted PEO produces more bubbles which come from cavitation and ultrasonic streaming induced by ultrasonic vibration. As the ultrasonic frequency increases, the size of cavitation bubbles decreases.
