# Supplementary material for: Effect of Ultrasonic Frequency on Structure and Corrosion Properties of Coating Formed on Magnesium Alloy via Plasma Electrolytic Oxidation
Source: Materials (Basel). 2023 Aug 2;16(15):5424. doi: 10.3390/ma16155424 (PMC10419714; doi:10.3390/ma16155424)
Supplement: Supplementary file 1 [file materials-16-05424-s001.zip › materials-2521542-supplementary.pdf]

Supplementray Materials

Title : Effect of ultrasonic frequency on structure and corrosion properties of coating formed on Mg alloy via plasma electrolytic oxidation

Authors: Siti Fatimah, Farah Hazmatulhaq, Yujun Sheng, Tri Suhartono, Jeong Moo Oh, Nisa Nashrah, Jee-Hyun Kang, and Young Gun Ko*

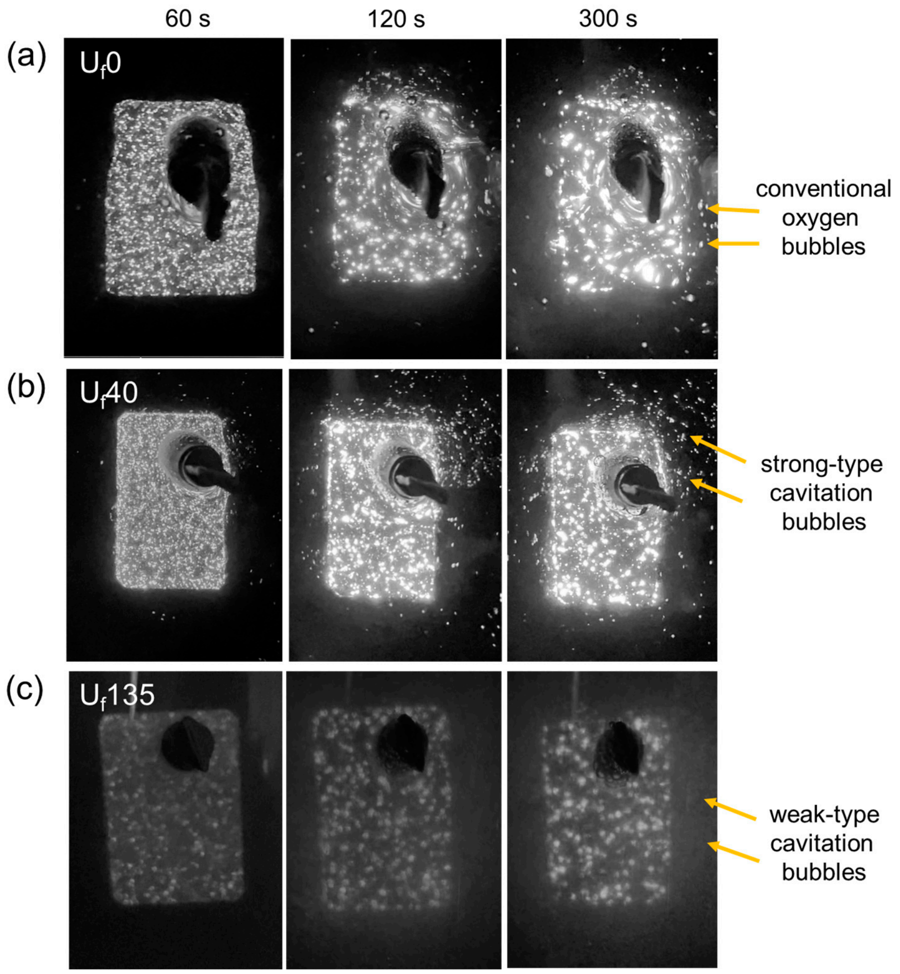

Figure S1. Optical images showing the plasma discharges appearance during PEO process under ultrasonic frequency of (a) 0, (b) 40, and (c) 135 kHz, respectively. Unlike conventional PEO, ultrasonic-assisted PEO produces more bubbles which come from cavitation and ultrasonic streaming induced by ultrasonic vibration. As the ultrasonic frequency increases, the size of cavitation bubbles decreases.
